# 2-Amino-9-[(1*S*,3*R*,4*S*)-4-hydr­oxy-3-hydroxy­methyl-2-methyl­enecyclo­pent­yl]-1,9-dihydro-6*H*-purin-6-one monohydrate

**DOI:** 10.1107/S1600536809032966

**Published:** 2009-08-26

**Authors:** Bin Jiang, Zhilu Liu

**Affiliations:** a Department of Pharmacy, Shandong Medical College, Jinan 250002, People’s Republic of China; b State Key Laboratory of Solid Lubrication, Lanzhou Institute of Chemical Physics, Chinese Academy of Sciences, Lanzhou 73000, People’s Republic of China

## Abstract

In the crystal of the title compound, C_12_H_15_N_5_O_3_·H_2_O, the component species are linked by N—H⋯N, N—H⋯O, O—H⋯N and O—H⋯O hydrogen bonds, forming a three-dimesnional network.

## Related literature

For background, see: Czarnik (2008 ▶).
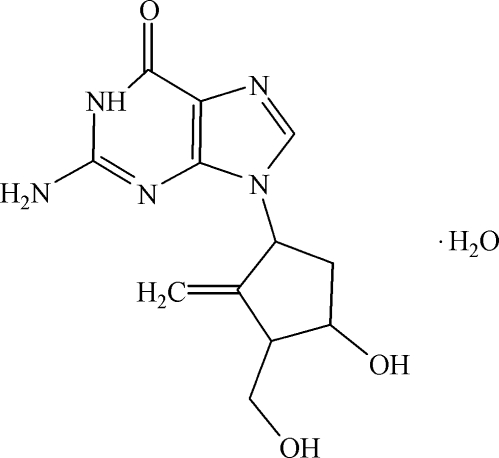


## Experimental

### 

#### Crystal data

C_12_H_15_N_5_O_3_·H_2_O
*M* *_r_* = 295.31
Orthorhombic, 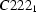

*a* = 6.9986 (10) Å
*b* = 11.6229 (10) Å
*c* = 33.932 (3) Å
*V* = 2760.1 (5) Å^3^
*Z* = 8
Mo *K*α radiation
μ = 0.11 mm^−1^
*T* = 273 K
0.12 × 0.10 × 0.08 mm

#### Data collection

Bruker APEXII CCD diffractometer
Absorption correction: multi-scan (*SADABS*; Bruker, 2004 ▶) *T*_min_ = 0.987, *T*_max_ = 0.991
6725 measured reflections
1377 independent reflections
1270 reflections with *I* > 2σ(*I*)
*R*_int_ = 0.030

#### Refinement

*R*[*F* ^2^ > 2σ(*F* ^2^)] = 0.030
*wR*(*F* ^2^) = 0.079
*S* = 1.00
1377 reflections
204 parameters
4 restraints
H atoms treated by a mixture of independent and constrained refinement
Δρ_max_ = 0.16 e Å^−3^
Δρ_min_ = −0.18 e Å^−3^

### 

Data collection: *APEX2* (Bruker, 2004 ▶); cell refinement: *SAINT-Plus* (Bruker, 2004 ▶); data reduction: *SAINT-Plus*; program(s) used to solve structure: *SHELXS97* (Sheldrick, 2008 ▶); program(s) used to refine structure: *SHELXL97* (Sheldrick, 2008 ▶); molecular graphics: *SHELXTL* (Sheldrick, 2008 ▶); software used to prepare material for publication: *SHELXTL*.

## Supplementary Material

Crystal structure: contains datablocks global, I. DOI: 10.1107/S1600536809032966/hb5032sup1.cif

Structure factors: contains datablocks I. DOI: 10.1107/S1600536809032966/hb5032Isup2.hkl

Additional supplementary materials:  crystallographic information; 3D view; checkCIF report

## Figures and Tables

**Table 1:** Hydrogen-bond geometry (Å, °)

*D*—H⋯*A*	*D*—H	H⋯*A*	*D*⋯*A*	*D*—H⋯*A*
O1—H1⋯N4^i^	0.82	2.04	2.857 (2)	172
O2—H2*A*⋯O1*W*^ii^	0.82	1.83	2.639 (3)	169
N3—H3*B*⋯O3^iii^	0.86	2.24	3.039 (3)	154
N5—H5*C*⋯N2^iii^	0.97 (3)	1.86 (3)	2.829 (3)	177 (3)
O1*W*—H2*W*⋯O1^iv^	0.819 (19)	2.113 (10)	2.900 (3)	161 (3)
O1*W*—H1*W*⋯O2^v^	0.821 (12)	2.000 (16)	2.783 (3)	159 (4)

Symmetry codes: (i) 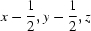; (ii) 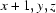; (iii) 
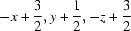
; (iv) 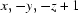; (v) 
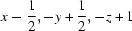
.

**Fig. 1.**
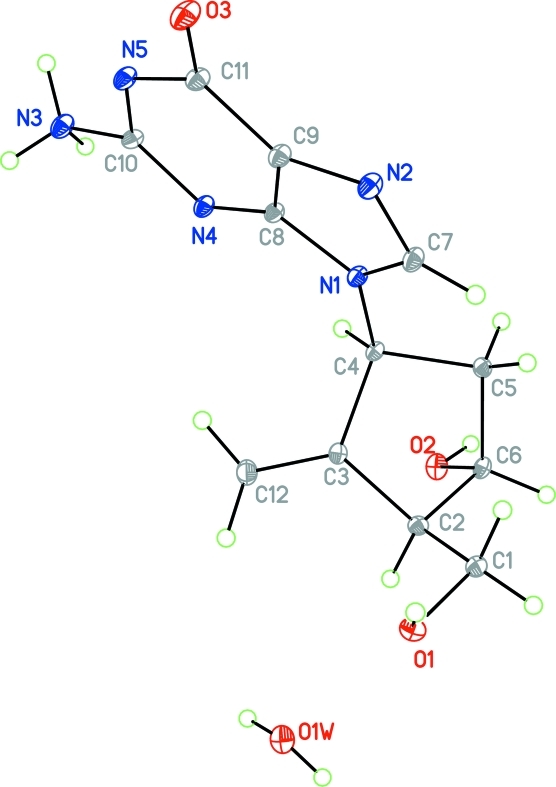
A view of (I) with displacement ellipsoids drawn at the 30% probability level.

**Fig. 2.**
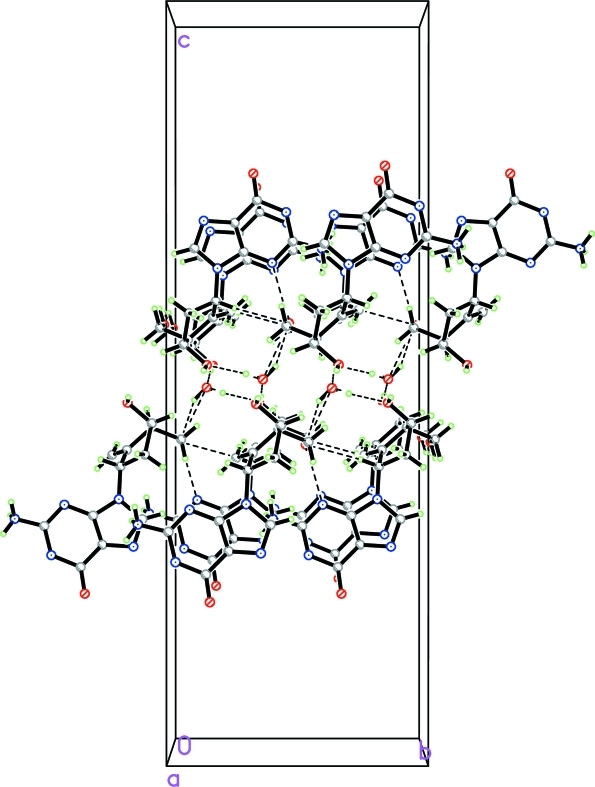
A view of (I) packing strcuture.

## supplementary crystallographic information

### Comment

The research of anti-hepatitis B virus (anti-HBV) drug has long been one of the serious diseases threatening human's health, and thus searching for effective medicines to cure such illness has led to significant interest over the past decades (Czarnik, 2008). In this article, we report the crystal structural characterization of 2-Amino-1,9-dihydro-9-[(1*S*,3*R*,4*S*)-4-hydroxy-3- (hydroxymethyl)-2-methylenecyclopentyl]-6*H*-purin-6-one.

As shown in figure 1, the asymmetrical unit contains one 2-Amino-1,9-dihydro-9-[(1*S*,3*R*,4*S*)-4-hydroxy-3-(hydroxymethyl)-2 -methylenecyclopentyl]-6*H*-purin-6-one and one water molecule. In addition, it is noteworthy that the multipoint hydrogen-bonding links also exist between the hydrogen atoms including N3—H3B···O3, 3.039 (3) Å; O1—H1···N4, 2.861 (2) Å; O2—H2A···O1W, 2.634 (2) Å; O1W—H2W···O1, 2.899 (3) Å; O1W—H1W···O2, 2.786 (3) Å; this may make a contribution to stabilizing the chain structure, shown in figure 2.

### Experimental

The reaction was performed in a 25-ml Teflon-lined stainless steel vessel. The powder of 2-amino-1,9-dihydro-9-[(1*S*,3*R*,4*S*)-4-hydroxy-3-(hydroxymethyl)-2 -methylenecyclopentyl]-6*H*-purin-6-one (1 mmol) in 5 ml water and 5 ml etanol was heated to 443 K and kept at this temperature for one day. Upon cooling, colourless blocks of (I) were recovered. Anal. Calc. for C_12_H_17_N_5_O_4_: C 48.76, H 5.08, N 23.70%; Found: 48.68, H 5.05, N 23.66%.

### Refinement

Anomalous dispersion was negligible and Friedel pairs were merged before refinement.

All hydrogen atoms bound to carbon were refined using a riding model with C—H = 0.93 (aryl), 0.97 (methylene) or 0.96 Å (methyl), and with *U*_iso_(H) = 1.2U_eq_(C) (aryl, methylene) or 1.5U_eq_(C) (methyl). The water H atoms were refined with restraints of O—H = 0.82 (1)Å and H···H = 1.38 (1)Å.

### Crystal data

**Table:**

C_12_H_15_N_5_O_3_·H_2_O	*F*(000) = 1248
*M**_r_* = 295.31	*D*_x_ = 1.421 Mg m^−^^3^
Orthorhombic, *C*222_1_	Mo *K*α radiation, λ = 0.71073 Å
Hall symbol: C 2c 2	Cell parameters from 1377 reflections
*a* = 6.9986 (10) Å	θ = 3.4–25.0°
*b* = 11.6229 (10) Å	µ = 0.11 mm^−^^1^
*c* = 33.932 (3) Å	*T* = 273 K
*V* = 2760.1 (5) Å^3^	Block, colorless
*Z* = 8	0.12 × 0.10 × 0.08 mm

### Data collection

**Table:**

Bruker APEXII CCD diffractometer	1377 independent reflections
Radiation source: fine-focus sealed tube	1270 reflections with *I* > 2σ(*I*)
graphite	*R*_int_ = 0.030
φ and ω scans	θ_max_ = 25.0°, θ_min_ = 3.4°
Absorption correction: multi-scan (*SADABS*; Bruker, 2004)	*h* = −8→8
*T*_min_ = 0.987, *T*_max_ = 0.991	*k* = −11→13
6725 measured reflections	*l* = −33→40

### Refinement

**Table:**

Refinement on *F*^2^	Primary atom site location: structure-invariant direct methods
Least-squares matrix: full	Secondary atom site location: difference Fourier map
*R*[*F*^2^ > 2σ(*F*^2^)] = 0.030	Hydrogen site location: inferred from neighbouring sites
*wR*(*F*^2^) = 0.079	H atoms treated by a mixture of independent and constrained refinement
*S* = 1.00	*w* = 1/[σ^2^(*F*_o_^2^) + (0.055*P*)^2^ + 0.4836*P*] where *P* = (*F*_o_^2^ + 2*F*_c_^2^)/3
1377 reflections	(Δ/σ)_max_ = 0.001
204 parameters	Δρ_max_ = 0.16 e Å^−^^3^
4 restraints	Δρ_min_ = −0.17 e Å^−^^3^

### Special details

**Table:**

Geometry. All e.s.d.'s (except the e.s.d. in the dihedral angle between two l.s. planes) are estimated using the full covariance matrix. The cell e.s.d.'s are taken into account individually in the estimation of e.s.d.'s in distances, angles and torsion angles; correlations between e.s.d.'s in cell parameters are only used when they are defined by crystal symmetry. An approximate (isotropic) treatment of cell e.s.d.'s is used for estimating e.s.d.'s involving l.s. planes.
Refinement. Refinement of *F*^2^ against ALL reflections. The weighted *R*-factor *wR* and goodness of fit *S* are based on *F*^2^, conventional *R*-factors *R* are based on *F*, with *F* set to zero for negative *F*^2^. The threshold expression of *F*^2^ > σ(*F*^2^) is used only for calculating *R*-factors(gt) *etc*. and is not relevant to the choice of reflections for refinement. *R*-factors based on *F*^2^ are statistically about twice as large as those based on *F*, and *R*- factors based on ALL data will be even larger.

### Fractional atomic coordinates and isotropic or equivalent isotropic displacement parameters (Å^2^)

**Table:**

	*x*	*y*	*z*	*U*_iso_*/*U*_eq_	
C1	0.5675 (3)	−0.0420 (2)	0.57539 (7)	0.0345 (5)	
H1A	0.5960	−0.1042	0.5573	0.041*	
H1B	0.6267	−0.0597	0.6005	0.041*	
C2	0.6493 (3)	0.0717 (2)	0.55918 (6)	0.0297 (5)	
H2	0.5838	0.0930	0.5347	0.036*	
C3	0.6333 (3)	0.16870 (18)	0.58921 (6)	0.0269 (5)	
C4	0.8242 (3)	0.18411 (18)	0.60945 (6)	0.0267 (5)	
H4	0.8800	0.2562	0.5999	0.032*	
C5	0.9461 (3)	0.0841 (2)	0.59303 (7)	0.0314 (5)	
H5A	1.0796	0.1060	0.5911	0.038*	
H5B	0.9355	0.0164	0.6096	0.038*	
C6	0.8611 (3)	0.0616 (2)	0.55207 (6)	0.0306 (5)	
H6	0.8961	−0.0146	0.5420	0.037*	
C7	0.7957 (4)	0.10135 (19)	0.67986 (7)	0.0364 (6)	
H7	0.7995	0.0242	0.6727	0.044*	
C8	0.8003 (3)	0.29145 (18)	0.67510 (6)	0.0293 (5)	
C9	0.7746 (4)	0.25703 (19)	0.71395 (7)	0.0338 (5)	
C10	0.7892 (3)	0.48170 (19)	0.68899 (6)	0.0327 (5)	
C11	0.7511 (4)	0.34291 (19)	0.74423 (7)	0.0353 (5)	
C12	0.4843 (4)	0.2341 (2)	0.59666 (9)	0.0445 (6)	
H12A	0.4935	0.2931	0.6151	0.053*	
H12B	0.3697	0.2215	0.5835	0.053*	
N1	0.8142 (3)	0.19086 (16)	0.65304 (5)	0.0310 (4)	
N2	0.7723 (3)	0.13654 (16)	0.71662 (6)	0.0395 (5)	
N3	0.7949 (3)	0.59520 (16)	0.67918 (6)	0.0424 (5)	
H3A	0.8106	0.6153	0.6550	0.051*	
H3B	0.7829	0.6468	0.6972	0.051*	
N4	0.8083 (3)	0.40206 (16)	0.66034 (5)	0.0331 (5)	
N5	0.7614 (3)	0.45538 (16)	0.72814 (5)	0.0362 (5)	
O1	0.3698 (2)	−0.03239 (15)	0.58020 (5)	0.0387 (4)	
H1	0.3405	−0.0525	0.6026	0.058*	
O2	0.9107 (3)	0.15191 (15)	0.52463 (5)	0.0422 (5)	
H2A	1.0233	0.1455	0.5183	0.063*	
O3	0.7248 (3)	0.32814 (15)	0.78032 (5)	0.0499 (5)	
O1W	0.2565 (3)	0.13435 (18)	0.49421 (6)	0.0500 (5)	
H2W	0.274 (5)	0.0919 (17)	0.4753 (5)	0.069 (12)*	
H1W	0.274 (7)	0.2029 (6)	0.4897 (8)	0.102 (16)*	
H5C	0.754 (5)	0.518 (2)	0.7470 (8)	0.080 (10)*	

### Atomic displacement parameters (Å^2^)

**Table:**

	*U*^11^	*U*^22^	*U*^33^	*U*^12^	*U*^13^	*U*^23^
C1	0.0340 (12)	0.0315 (13)	0.0380 (13)	−0.0029 (10)	−0.0012 (10)	−0.0008 (11)
C2	0.0331 (12)	0.0313 (12)	0.0246 (10)	−0.0001 (10)	−0.0015 (9)	0.0007 (9)
C3	0.0304 (11)	0.0236 (11)	0.0269 (10)	0.0008 (9)	0.0050 (9)	0.0052 (9)
C4	0.0325 (11)	0.0223 (11)	0.0252 (10)	−0.0022 (9)	0.0036 (8)	−0.0009 (9)
C5	0.0267 (11)	0.0321 (12)	0.0355 (12)	0.0022 (10)	0.0017 (9)	−0.0033 (10)
C6	0.0371 (12)	0.0267 (11)	0.0282 (11)	0.0004 (10)	0.0072 (9)	−0.0033 (9)
C7	0.0539 (15)	0.0242 (11)	0.0312 (12)	0.0028 (11)	0.0004 (11)	0.0012 (9)
C8	0.0357 (12)	0.0269 (11)	0.0255 (11)	−0.0007 (10)	0.0004 (9)	−0.0028 (9)
C9	0.0461 (13)	0.0291 (11)	0.0260 (11)	−0.0006 (11)	0.0023 (11)	0.0004 (9)
C10	0.0390 (13)	0.0317 (12)	0.0272 (11)	−0.0021 (11)	0.0011 (10)	−0.0027 (9)
C11	0.0455 (13)	0.0346 (11)	0.0258 (12)	−0.0019 (12)	0.0012 (10)	0.0007 (9)
C12	0.0395 (14)	0.0359 (14)	0.0580 (17)	0.0044 (12)	0.0021 (12)	−0.0019 (12)
N1	0.0416 (11)	0.0256 (9)	0.0260 (9)	−0.0015 (9)	0.0005 (8)	−0.0019 (8)
N2	0.0594 (13)	0.0299 (10)	0.0292 (10)	−0.0009 (10)	0.0029 (10)	0.0047 (8)
N3	0.0707 (15)	0.0281 (10)	0.0284 (10)	−0.0006 (11)	0.0061 (10)	−0.0004 (8)
N4	0.0477 (12)	0.0273 (10)	0.0243 (9)	−0.0005 (9)	0.0033 (9)	−0.0017 (8)
N5	0.0544 (12)	0.0298 (10)	0.0242 (9)	−0.0007 (10)	0.0036 (9)	−0.0041 (8)
O1	0.0332 (9)	0.0438 (10)	0.0389 (9)	−0.0070 (8)	0.0015 (7)	0.0058 (8)
O2	0.0431 (10)	0.0434 (10)	0.0401 (9)	0.0010 (8)	0.0184 (8)	0.0074 (8)
O3	0.0824 (14)	0.0433 (10)	0.0239 (8)	−0.0004 (10)	0.0077 (8)	0.0016 (7)
O1W	0.0490 (11)	0.0500 (12)	0.0512 (12)	0.0011 (11)	0.0141 (9)	0.0074 (10)

### Geometric parameters (Å, °)

**Table:**

C1—O1	1.398 (3)	C8—N4	1.381 (3)
C1—C2	1.542 (3)	C8—C9	1.389 (3)
C1—H1A	0.9700	C8—N1	1.392 (3)
C1—H1B	0.9700	C9—N2	1.403 (3)
C2—C6	1.506 (3)	C9—C11	1.442 (3)
C2—C3	1.524 (3)	C10—N4	1.349 (3)
C2—H2	0.9800	C10—N3	1.361 (3)
C3—C12	1.316 (3)	C10—N5	1.377 (3)
C3—C4	1.513 (3)	C11—O3	1.250 (3)
C4—N1	1.483 (2)	C11—N5	1.418 (3)
C4—C5	1.546 (3)	C12—H12A	0.9300
C4—H4	0.9800	C12—H12B	0.9300
C5—C6	1.534 (3)	N3—H3A	0.8600
C5—H5A	0.9700	N3—H3B	0.8600
C5—H5B	0.9700	N5—H5C	0.97 (3)
C6—O2	1.445 (3)	O1—H1	0.8200
C6—H6	0.9800	O2—H2A	0.8200
C7—N2	1.323 (3)	O1W—H2W	0.819 (19)
C7—N1	1.388 (3)	O1W—H1W	0.821 (12)
C7—H7	0.9300		
			
O1—C1—C2	109.91 (19)	N2—C7—N1	113.4 (2)
O1—C1—H1A	109.7	N2—C7—H7	123.3
C2—C1—H1A	109.7	N1—C7—H7	123.3
O1—C1—H1B	109.7	N4—C8—C9	128.1 (2)
C2—C1—H1B	109.7	N4—C8—N1	125.74 (19)
H1A—C1—H1B	108.2	C9—C8—N1	106.11 (19)
C6—C2—C3	103.73 (19)	C8—C9—N2	110.5 (2)
C6—C2—C1	110.8 (2)	C8—C9—C11	119.4 (2)
C3—C2—C1	111.62 (18)	N2—C9—C11	130.0 (2)
C6—C2—H2	110.2	N4—C10—N3	119.1 (2)
C3—C2—H2	110.2	N4—C10—N5	123.8 (2)
C1—C2—H2	110.2	N3—C10—N5	117.10 (19)
C12—C3—C4	123.0 (2)	O3—C11—N5	120.7 (2)
C12—C3—C2	127.9 (2)	O3—C11—C9	128.3 (2)
C4—C3—C2	109.04 (18)	N5—C11—C9	110.97 (18)
N1—C4—C3	114.69 (17)	C3—C12—H12A	120.0
N1—C4—C5	115.16 (18)	C3—C12—H12B	120.0
C3—C4—C5	103.58 (17)	H12A—C12—H12B	120.0
N1—C4—H4	107.7	C7—N1—C8	105.70 (17)
C3—C4—H4	107.7	C7—N1—C4	128.21 (18)
C5—C4—H4	107.7	C8—N1—C4	125.75 (18)
C6—C5—C4	103.91 (17)	C7—N2—C9	104.25 (19)
C6—C5—H5A	111.0	C10—N3—H3A	120.0
C4—C5—H5A	111.0	C10—N3—H3B	120.0
C6—C5—H5B	111.0	H3A—N3—H3B	120.0
C4—C5—H5B	111.0	C10—N4—C8	111.93 (18)
H5A—C5—H5B	109.0	C10—N5—C11	125.68 (18)
O2—C6—C2	106.42 (19)	C10—N5—H5C	118 (2)
O2—C6—C5	111.52 (19)	C11—N5—H5C	116 (2)
C2—C6—C5	102.89 (18)	C1—O1—H1	109.5
O2—C6—H6	111.8	C6—O2—H2A	109.5
C2—C6—H6	111.8	H2W—O1W—H1W	115 (2)
C5—C6—H6	111.8		

### Hydrogen-bond geometry (Å, °)

**Table:**

*D*—H···*A*	*D*—H	H···*A*	*D*···*A*	*D*—H···*A*
O1—H1···N4^i^	0.82	2.04	2.857 (2)	172
O2—H2A···O1W^ii^	0.82	1.83	2.639 (3)	169
N3—H3B···O3^iii^	0.86	2.24	3.039 (3)	154
N5—H5C···N2^iii^	0.97 (3)	1.86 (3)	2.829 (3)	177 (3)
O1W—H2W···O1^iv^	0.82 (2)	2.11 (1)	2.900 (3)	161 (3)
O1W—H1W···O2^v^	0.82 (1)	2.00 (2)	2.783 (3)	159 (4)

Symmetry codes: (i) *x*−1/2, *y*−1/2, *z*; (ii) *x*+1, *y*, *z*; (iii) −*x*+3/2, *y*+1/2, −*z*+3/2; (iv) *x*, −*y*, −*z*+1; (v) *x*−1/2, −*y*+1/2, −*z*+1.
